# Autologous stem cell therapy for peripheral arterial disease: a systematic review and meta-analysis of randomized controlled trials

**DOI:** 10.1186/s13287-019-1254-5

**Published:** 2019-05-21

**Authors:** Wei Gao, Dawei Chen, Guanjian Liu, Xingwu Ran

**Affiliations:** 1 Diabetic Foot Care Center, Department of Endocrinology and Metabolism, West China Hospital, Sichuan University, Chengdu, 610041 People’s Republic of China; 2 Health Management Center, West China Hospital, Sichuan University, Chengdu, 610041 People’s Republic of China; 3 Chinese Cochrane Centre, Chinese EBM Centre, West China Hospital, Sichuan University, Chengdu, 610041 People’s Republic of China

**Keywords:** Peripheral arterial disease, Critical limb ischemia, Autologous, Stem cells, Implantation, Systematic review, Meta-analysis, Randomized controlled trials

## Abstract

**Background:**

Peripheral arterial disease (PAD) is a common cause of disability and mortality. The reconstruction of blood circulation presents to be the key to treatment, which can be achieved by surgery and interventional therapy. Since 40% patients have lost the chance for the therapy, a new method is needed to reduce the amputation and mortality rate for “no-option” patients. The objective of our systematic review and meta-analysis was to evaluate the efficacy and safety of autologous implantation of stem cells in patients with PAD critically, compared with active controls and placebo.

**Methods:**

Randomized controlled trials (RCTs) of autologous implantation of stem cells compared with placebo and control for PAD were included. Electronic medical databases including MEDLINE, Embase, the Cochrane Central Register of Controlled Trials (CENTRAL), the Chinese Biomedical Literature Database, China National Knowledge Infrastructure (CNKI), and ClinicalTrials.gov were searched from initial period to September 2018. Independently, two reviewers screened citations, extracted data, and assessed the risk of bias according to the criteria of the Cochrane handbook. The quality of evidence was evaluated by GRADE evidence profile. The primary outcomes consisted of amputation rate, major amputation rate, ulcer healing rate, and side effects. The second outcomes included ankle-brachial index (ABI), transcutaneous oxygen tension (TcO2), pain-free walking distance (PFWD), and rest pain score. Statistical analysis was conducted via RevMan 5.3 and Stata 12.0.

**Results:**

According to the twenty-seven RCTs, 1186 patients and 1280 extremities were included and the majority of studies showed a high risk of bias. Meta-analysis indicated that autologous stem cell therapy was more effective than conventional therapy on the healing rate of ulcers [OR = 4.31 (2.94, 6.30)]. There was also significant improvement in ABI [MD = 0.13 (0.10, 0.17)], TcO2 [MD = 0.13 (0.10, 0.17)], and PFWD [MD = 178.25 (128.18, 228.31)] while significant reduction was showed in amputation rate [OR = 0.50 (0.36, 0.69)] and rest pain scores [MD = − 1.61 (− 2.01, − 1.21)]. But the result presented no significant improvement in major limb salvage [0.66 (0.42, 1.03)]. Besides, stem cell therapy could reduce the amputation rate [OR = 0.50 (0.06, 0.45] and improve the ulcer healing rate [OR = 4.34 (2.96, 6.38] in DM subgroup. Eight trials reported the side effects of autologous stem cell therapy, and no serious side effects related to stem cells were reported. GRADE evidence profile showed all the quality evidence of outcomes were low.

**Conclusions:**

Based on the review, autologous stem cell therapy may have a positive effect on “no-option” patients with PAD, but presented no significant improvement in major limb salvage. However, the evidence is insufficient to prove the results due to high risk of bias and low-quality evidence of outcomes. Further researches of larger, randomized, double-blind, placebo-controlled, and multicenter trials are still in demand.

**Electronic supplementary material:**

The online version of this article (10.1186/s13287-019-1254-5) contains supplementary material, which is available to authorized users.

## Background

Peripheral arterial diseases (PAD), as a member of arteriosclerosis, mostly occur in lower extremity arteries. The morbidity of PAD generally ranged from 3 to 10%, but among the people over 60 years old, it can reach above 15% and it upregulates with aging [1]. PAD is one of the most serious complications in patients with diabetes mellitus (DM), and the overall prevalence is 21.2% in China [2]. If not properly treated in the early stage, it is very possible for the patient to suffer from critical limb ischemia (CLI) causing rest pain, ulcer, necrosis, and finally leading to amputation. The rate of amputation among PAD patients is about 1.6~4.1% with even much higher cardiovascular event incidence and mortality [3–5].

Patients with CLI are commonly treated conventionally at an early stage, such as risk factor control, exercise training, utilizing antiplatelet drugs, and vasodilator [6–12]. But the reconstruction of the blood circulation, which can be achieved by surgery and interventional therapy, presented to be the key to the treatment [13, 14]. A 5-year survival rate which was less than 50% determined a worrisome prognosis. And when both surgery and interventional therapy is not feasible, amputation may be the last choice of the patients. However, amputation has a high rate of mortality about 25~50%, of which 5~20% in perioperational period, and the re-amputation rate is up to 30% [15]. The risk is significantly raising in patients with DM, for the segmental and diffuse arterial disease as well as the higher risk of cardiovascular event. Since 40% patients have missed the chance for surgery or interventional therapy [16], a new method is in great demand to reduce the amputation and mortality rate for “no-option” patients.

Autologous stem cell therapy is gradually known as a new therapy. Asahara isolated endothelial progenitor cells (EPCs) from blood in 1997 [17]. EPCs are a type of adult stem cells, derived from adult bone marrow and is mainly found in the embryo, adult peripheral blood, umbilical cord blood, and bone marrow. EPCs can develop into endothelial cells and then promote revascularization. Methods for isolation of EPCs include magnetic bead selection, density gradient centrifugation, and differential adhesion method and so on. Many animal trials found improved blood flows in ischemic limbs after stem cell implantation [18–21]. Afterward, the therapies of stem cells have been applied to patients with PAD. The first trial in human called therapeutic angiogenesis using cell transplantation (TACT) was performed in Japan [22]. Since then, a growing body of evidence suggested that autologous stem cell therapy was more effective than standard care/conventional treatment for PAD [23]. Former systematic review pooled analysis of both randomized controlled trials (RCTs) and non-RCTs; however, studies of different designs cannot be assessed in unification. Therefore, in the present study, we updated the systematic review to evaluate the efficacy and safety of autologous implantation of stem cells for PAD.

## Methods

We followed the recommendations from the Cochrane Collaboration for systematic review and meta-analysis of RCTs and reported according to preferred reporting items for systematic reviews and meta-analyses (PRISMA) statements [24].

### Inclusion criteria and searching strategies

We searched RCTs involving patients with PAD who were treated with autologous implantation of all kinds of stem cells from electronic medical databases including MEDLINE, Embase, the Cochrane Central Register of Controlled Trials (CENTRAL), the Chinese Biomedical Literature Database, China National Knowledge Infrastructure (CNKI), and ClinicalTrials.gov from initial period to September 2018. The MeSH terms were outlined in Additional file 1: Table S1.

### Data extraction and bias assessment

Two investigators selected the studies and extracted data from studies independently. Controversy was resolved by discussion with a third investigator. Extracted data included basic information (author name, study year, country, sample size, design of study, follow-up time), characteristics of patients (sex, age, stage of PAD), methods, intervention details (type and number of stem cells, transplantation routine, intervention in control group), outcomes, and side effect. The bias of the trials included in our study was assessed according to the Cochrane Handbook for Interventions [25]. The components included allocation sequence generation, allocation concealment, blinding of participants, caregivers, outcome assessors and outcome adjudicators, incomplete outcome data, selective outcome reporting, and other sources of bias. For each item, studies were categorized as high, low, or unclear risk of bias.

### Observation index

The primary outcomes consisted of amputation rate, major amputation rate, ulcer healing rate, and side effect. The second outcomes were ankle-brachial index (ABI), transcutaneous oxygen tension (TcO_2_), rest pain score, and pain-free walking distance (PFWD).

### Statistical analysis

We performed a meta-analysis of all RCTs using the data from the cell therapy group and control group. Statistical analysis was conducted via RevMan 5.3 and Stata 12.0. Continuous and dichotomous outcome variables were respectively described as mean difference (MD) and odds ratios (OR) with 95% confidence intervals (CI), which were derived from Inverse Variance and Mantel-Haenszel estimate and summarized by Forest plots. Heterogeneity among studies was evaluated by the *I*^2^ parameter and chi-squared tests. Fixed effect model was used for meta-analysis when *I*^2^ values < 50% and random effect model when *I*^2^ values ≥ 50% as heterogeneity indicated. Incomplete outcome data were analyzed by intention to treat analysis. Sensitivity analyses were conducted to examine the difference between random and fixed effects model as to their effect measures such as OR, relative risk (RR), and risk difference (RD). We explored the publication bias by funnel plots (when the number of included studies more than 9) and Egger’s test for continuous endpoints and Harbord’s test for dichotomous endpoints.

The GRADE approach was used to evaluate the quality of evidence of each outcome, which was classified as high, moderate, low, and very low after the all-round assessment of study limitations, inconsistency, imprecision, indirectness, and publication bias [26].

## Results

### Study selection and characteristics

**Fig. 1:**
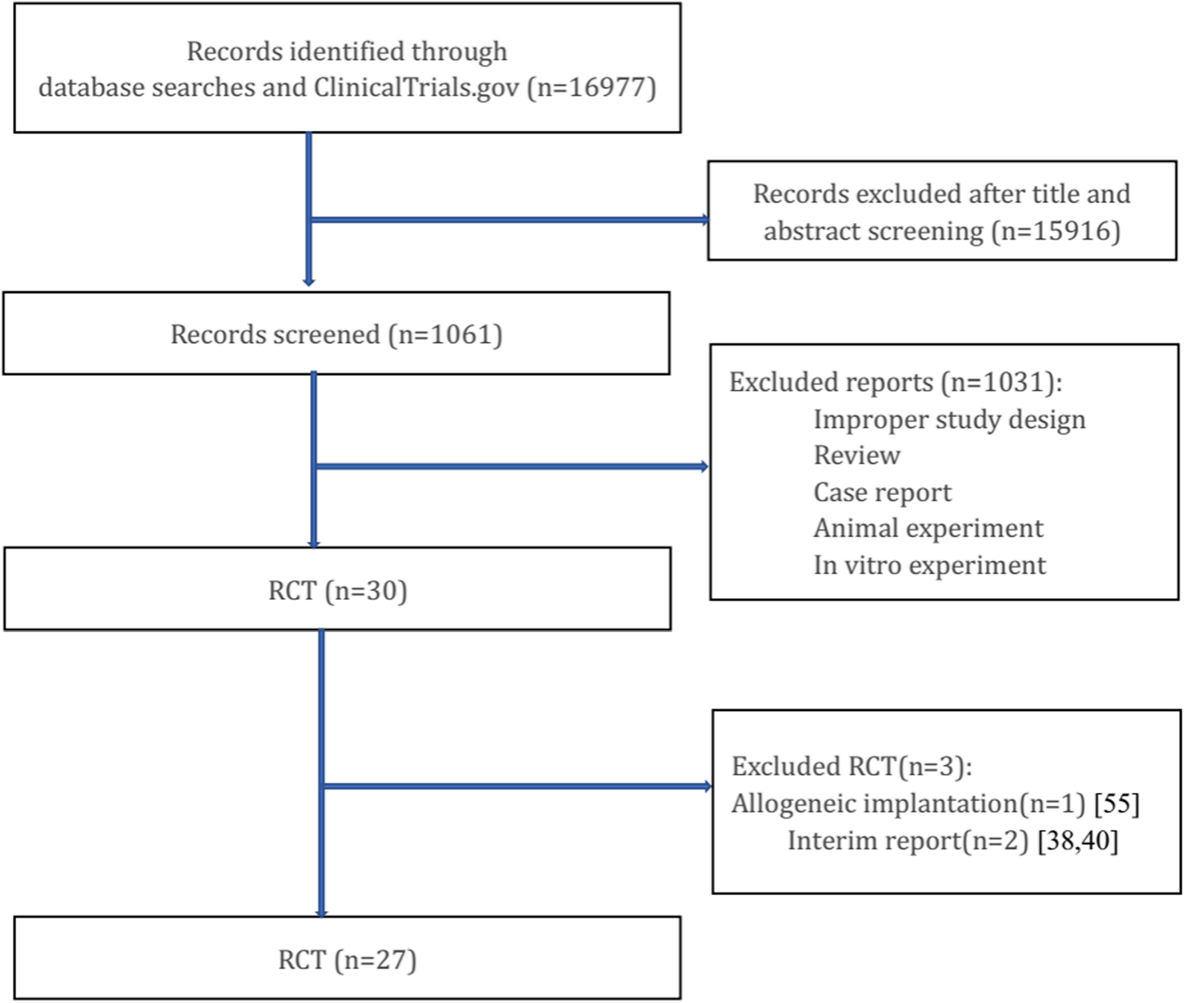
Flow chart of selection of studies

Among the 16,977 studies, 27 RCTs [22, 27–54] involving 1186 patients and 1280 limbs were included in our systematic review. The inclusion and exclusion flow was listed in flow Fig. 1. Among the 27 RCTs, 16 studies were from Asians [22, 27, 29–35, 37, 43–45, 48, 50, 54], 7 [28, 36, 38, 46, 49, 52, 53] from Europeans, and 4 [39–42, 47, 51] from Americans. Patients in the trials were identified as PAD or diabetic foot (DF) with different classifications. Stem cells, including BMMSCs, BMMNCs, BMAC, PBMNC, CD34+ cells, VesCell, PBMCs, and CD133+ cells, were transplanted by intramuscular injection [22, 27–37, 39–51, 53, 54] or intra-arterial injection [28, 38, 52]. The average follow-up time was 4.7 months (1–36 m). Details of studies were listed in Table 1.

**Table 1:** Characteristics of clinical trials included in the systematic review

Author (year)	Country	Subjects	No. of patients		No. of Limbs		Average age (years)		No. of male		Treatment strategy		Follow-up (months)
			Treatment	Control	Treatment	Control	Treatment	Control	Treatment	Control	Treatment (type, no., route)	Control	
Tateishi-Yuyama et al. (2002) [22]	Japan	CLI Fontaine III–IV	22	22	22	22	–	–	–	–	BMMSCs 1.5 ± 0.6 × 10^9^ IM	No mobilized PBMNCs	6
Huang et al. (2005) [27]	China	DM with CLI Fontaine III–IV	14	14	23	24	71.1	70.9	9	9	PBMNCs 3 × 10^9^ IM	Conventional therapy	3
Barć et al. (2006) [28]	Poland	CLI	14	15	14	15	–	–	–	–	BMMSCs – IM (14 patients) IA (4 patients)	Conventional therapy	6
Arai et al. (2006) [29]	Japan	CLI Fontaine III or IV	13	12	13	12	62	68	11	7	BMMSCs (1–3) × 10^9^ IM	Conventional therapy	1
Zhang et al. (2007) [30]	China	DF Wagner1–3	31	30	31	30			13	12	BMMSCs – IM	Conventional therapy	1
Lu et al. (2008) [31]	China	DM with CLI FontaineII–IV	25	25	25	25	66.6	65.5	11	15	BMMSCs 7.32 × 10^8^–5.61 × 10^9^ IM	Conventional therapy	3
Dash et al. (2009) [32]	India	Buerger’s disease and DF (with ulcer)	12	12	12	12	–	–	–	–	BM MSC 5.04–7.26 × 10^6^ IM	Conventional therapy	3
Chen et al. (2009) [33]	China	DF Wagner2–4	22	18	22	18	65.8	63.5	–	–	BM MSC – IM	Conventional therapy	1
Gan et al. (2009) [34]	China	DF Wagner1–4	15	15	28	30	–	–	–	–	BM MSC (1.35–9.36) × 10^8^ IM	Conventional therapy	3–12
Shi et al. (2009) [35]	China	DM with PAD	25	25	25	25	35-75		23		BMSCs – IM	Conventional therapy	3
Procházka et al. (2010) [36]	Czech Republic	CLI with foot ulcer	42	54	42	54	66.2 ± 10.6	64.1 ± 8.6	36	42	ABMSC 0.7–3.83 × 10^9^ IM	Conventional therapy	3–4
Wen and Huang (2010) [37]	China	CLI Fontaine II–V	30	30	112		60.8 ± 8.6	61.7 ± 8.3	20	19	PBSCs 3 × 10^9^ IM	Conventional therapy	3–36
Walter et al. (2011) [38]	Germany	CLI	19	21	19	21	64.4 ± 15	64 ± .516	16	13	BM MNC 1.53 × 10^8^ IA	Placebo	3
Iafrati et al. (2011) [39] Benoit et al. (2011) [40]	America	CLI Rutherford4–5	34	14	34	14	72.5	65.7	23	7	BMAC 3.23 × 10^9^ IM	Placebo	3–6
Powell et al. (2011) [41] Powell et al (2012) [42]	America	CLI	48	24	48	24	69.2 ± 13.2	67.3 ± 11.6	34	14	Ixmyelocel-T – IM	Placebo	6–12
Lu et al. (2011) [43]	China	DM with CLI	20 (BMMSCs) 21 (BMMNCs)	41	20 (BMMSCs) 21 (BMMNCs)	41	–	–	–	–	BMMSCs 9.3 × 10^8^ BMMNs 9.6 × 10^8^ IM	Placebo	6
Guan et al. (2011) [44]	China	DF Wagner1–4	39	40	78	80	69 ± 16		45		BM-MNC 1.27~8.95) × 108 IM	Conventional therapy	6–36
Jain et al. (2011) [45]	India	chronic lower limb wounds in DM	25	23	25	23	54	58	–	–	BMSCs – IM	Conventional therapy	3
Ozturk et al. (2012) [46]	Turkey	DM with CLI Fontaine III–IV	20	20	20	20	79.9 ± 9.2	70.8 ± 8.8	16	13	PBMNC 9.92 × 10^8^–1.24 × 10^9^ IM	Conventional therapy	3
Losordo et al. (2012) [47]	America	CLI Rutherfod 4–5	7(LD) 9(HD)	12	7(LD) 9(HD)	12	61.8 ± 13.9(LD) 69.7 ± 10.9(HD)	67.1 ± 14.2	5(LD) 8(HD)	6	PMCD34+ 0.1/Kg (LD) 1/Kg (HD) IM	Placebo	12
Li et al. (2013) [48]	China	CLI	29	29	29	29	61 ± 9	63 ± 10	22	23	BM-MNC – IM	Placebo	6
Szabó et al. (2013) [49]	Hungary	Fontaine III-IV	10	10	10	10	60.6 ± 8.9	63.0 ± 12.0	8	5	VesCell 6.64 × 10^7^ IM	conventional therapy	3–24
Mohammadzadeh et al. (2013) [50]	Iran	DM with CLI	7	14	7	14	63.5 ± 7.8	64.2 ± 7.8	–	–	PBMCs 0.9–1.2 × 10^9^ IM	Placebo	3
Raval et al. (2014) [51]	America	CLI	7	3	7	3	65	85	6	2	PBCD133+/PLA 5 × 10^7^–4 × 10^8^ IM	Placebo	12
Teraa et al. (2015) [52]	Netherlands	CLI Fontaine IIb-IV	81	79	81	79	69	65	57	51	BMMNC 6.57 × 10^8^ IA	Placebo	6
Skóra et al. (2015) [53]	Poland	CLI Fontaine IV	16	16	16	16	66.76	68.3	11	10	BM MNC+VEGF 0.77–3.83 × 10^9^ IM	Pentoxifylline	3
Lu et al. (2016) [54]	China	DM with PAD	20	21	20	21	67.2		27		PBSCs – IM	Conventional therapy	6

*BMMNC* bone marrow mononuclear cells, *PBMNC* peripheral blood mononuclear cells, *BMAC* bone marrow aspirate concentrate, *BMMSC* bone marrow mesenchymal stem cells, *ABMSC* autologous bone marrow stem cells, *VesCell* peripheral blood-derived autologous angiogenic cell precursors, *IM* intramuscular, *IA* intraarterial, *LD* low dose, *HD* high dose, *VEGF* vascular endothelial growth factor

### Risk of bias

**Fig. 2:**
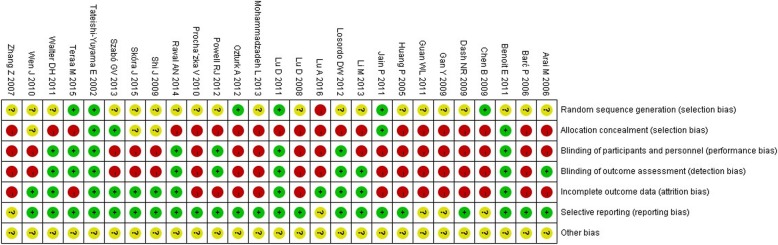
Risk of bias summary

**Fig. 3:**
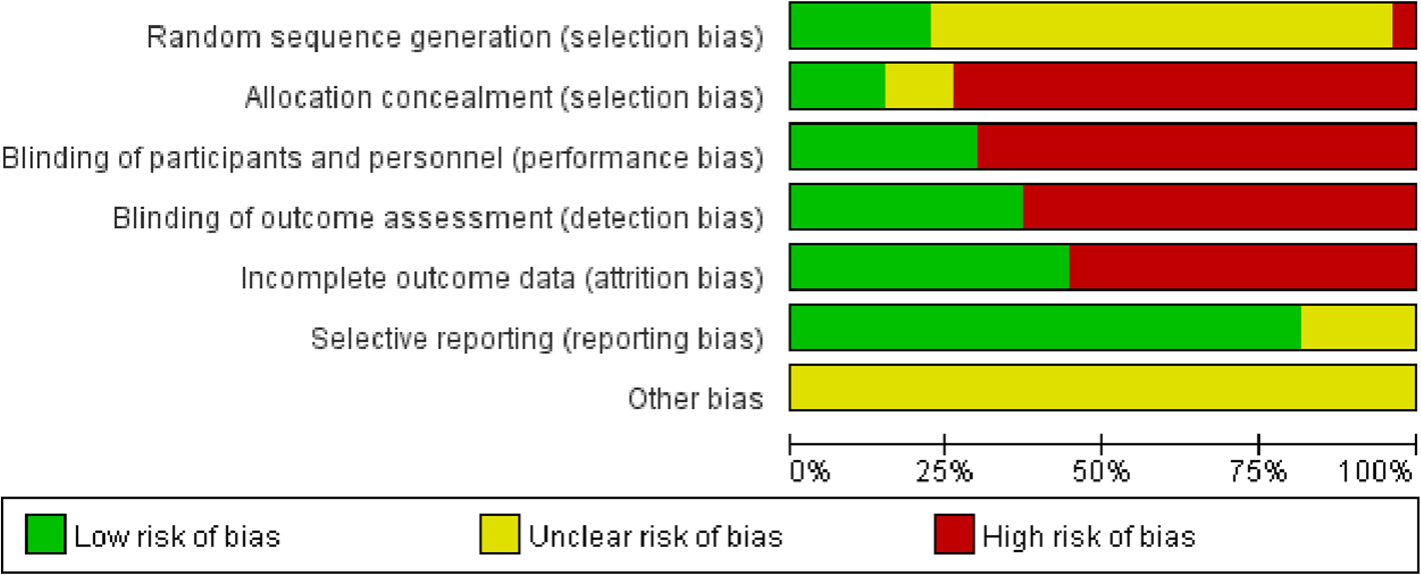
Risk of bias graph

According to Cochrane Handbook, each risk of bias item for each included RCTs and each risk of bias item of all included RCTs were presented in Figs. 2 and 3. The figures showed high risk of bias mainly resulted from the lack of allocation concealment, absent blinding, and incomplete outcome data. Among the 27 RCTs, only 6 (22.2%) studies [22, 33, 43, 45, 46, 52] adequately generated the randomization sequence, 4 (14.8%) [22, 40, 45, 49] concealed allocation, 8 [22, 38, 40, 42, 43, 47, 51, 52] (29.6%) blinding of participants and personnel, and 10 (37.0%) [22, 29, 38, 40, 42, 43, 47, 48, 51, 52] blinding of outcome assessment. Twelve (44.4%) [22, 35, 37, 38, 40, 43, 47–49, 51, 53, 54] trials had no incomplete outcome data, and 22 (81.5%) [22, 27–29, 31, 32, 35–38, 40, 42, 43, 45–53] were free of selective outcome reporting.

### Amputation rate

**Fig. 4:**
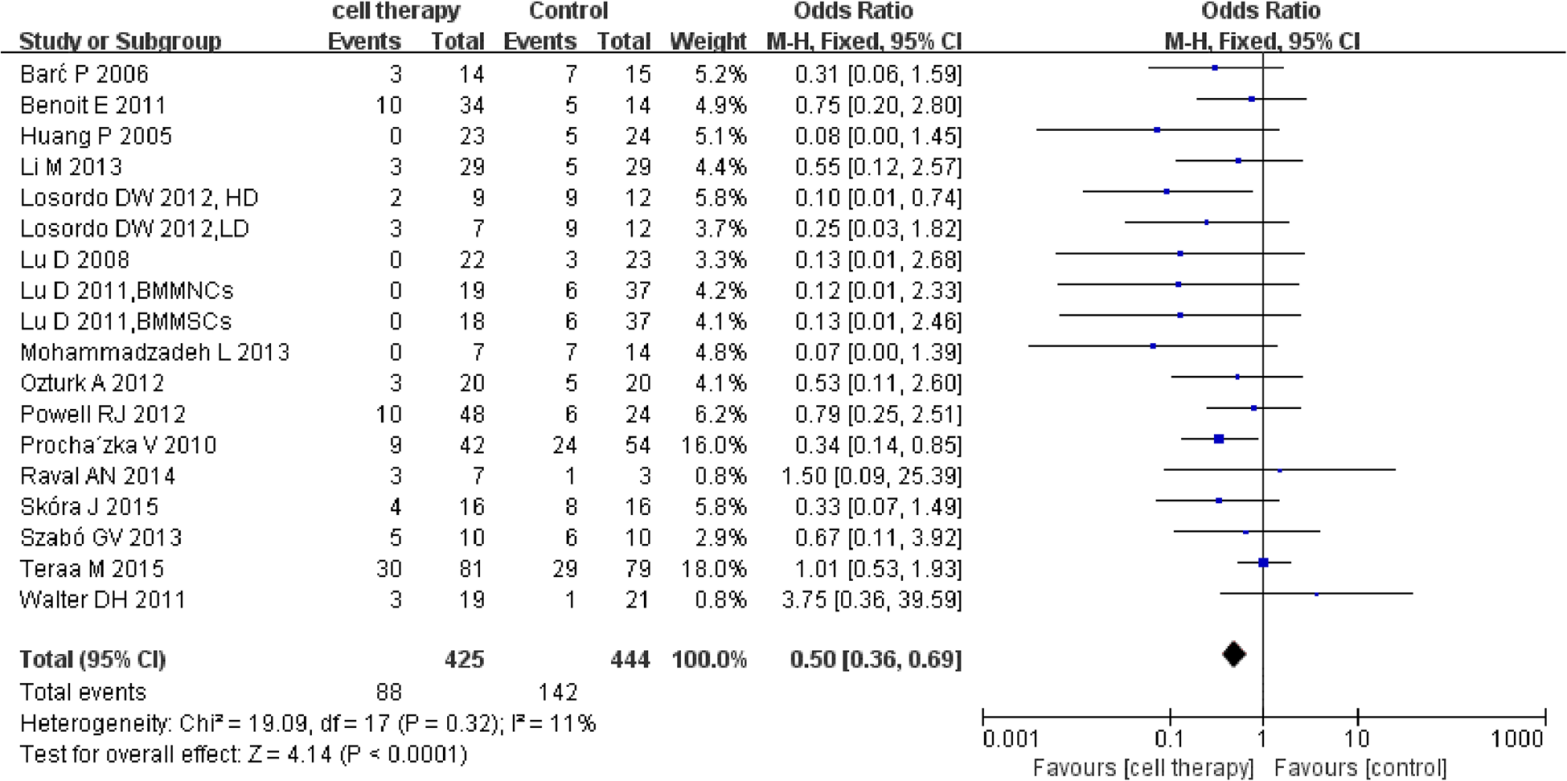
Forest plot showing the effect of stem cell therapy on amputation rate

Among the 27 RCTs, 16 trials [27, 28, 31, 36, 38, 40, 42, 43, 46–53] reported the detailed amputation rate. The meta-analysis showed a lower amputation rate in cell therapy group compared with control (88/425 vs 142/444; OR 0.50, 95% CI 0.36 to 0.69, *I*^2^ = 11%)(Fig. 4).

### Major amputation rate

**Fig. 5:**
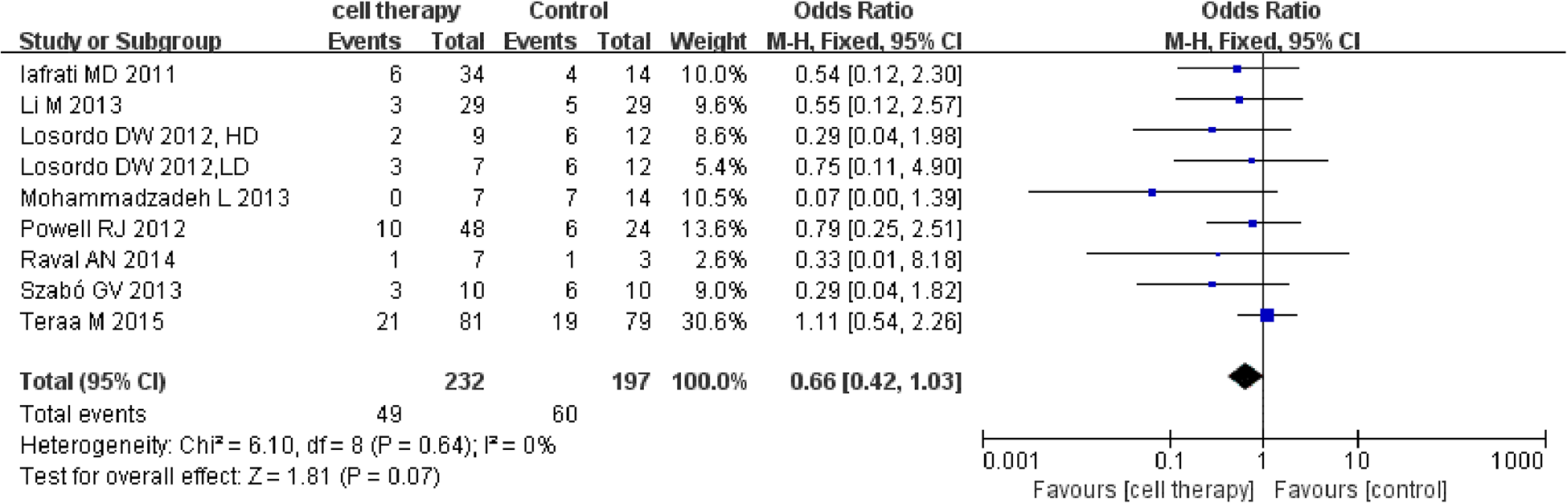
Forest plot showing the effect of stem cell therapy on major amputation rate

Eight studies [39, 42, 47–52] reported the detailed major amputation rate. The meta-analysis showed a lower major amputation rate in the stem cell therapy group than control but with no statistical significance (49/232 vs. 60/197; OR 0.66, 95% CI 0.42 to 1.03, *I*^2^ = 0%) (Fig. 5).

### Ulcer healing rate

**Fig. 6:**
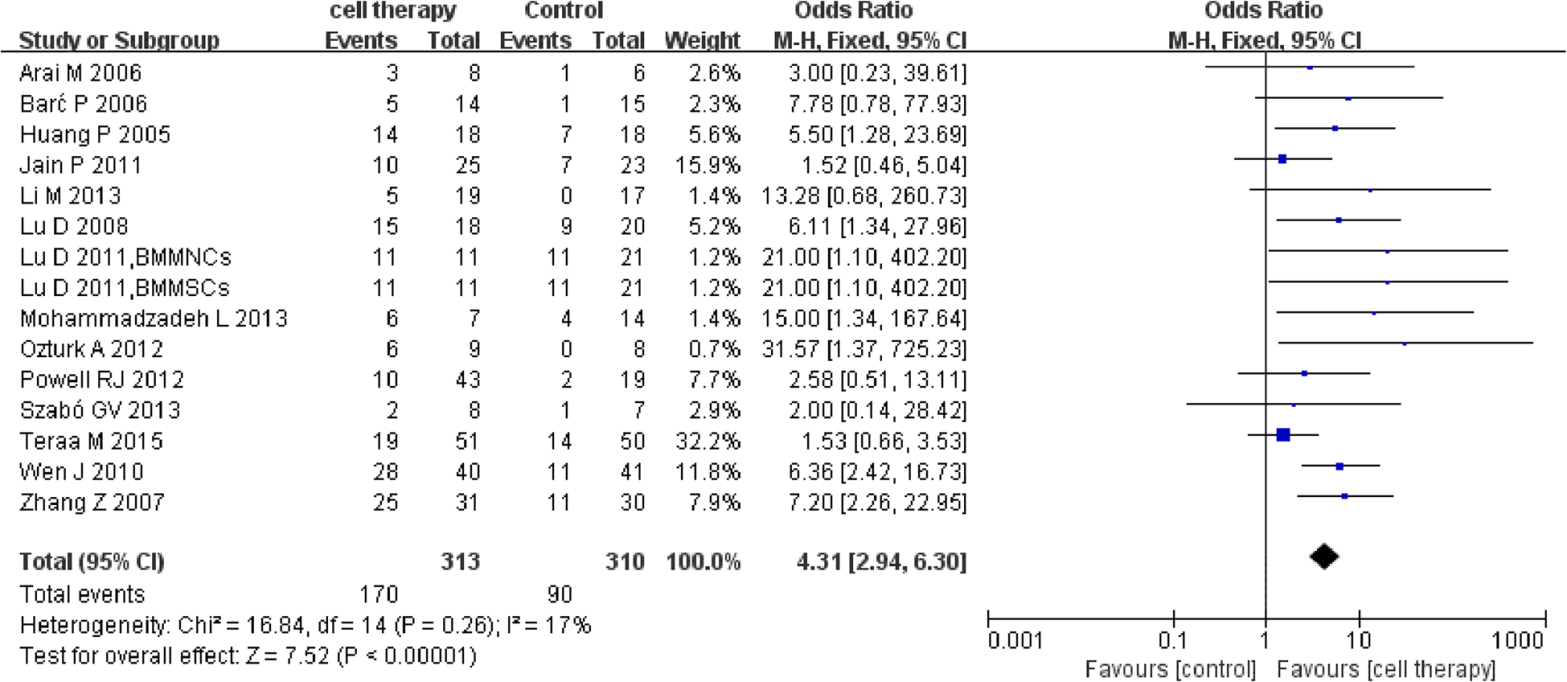
Forest plot showing the effect of stem cell therapy on ulcer healing rate

Fourteen studies [27–31, 37, 42, 43, 45, 46, 48–50, 52] reported the detailed ulcer healing rate. The meta-analysis showed a higher ulcer healing rate in the cell therapy group compared with control (170/313 vs 90/310; OR 4.31, 95% CI 2.94 to 6.30, I^2^ = 17%) (Fig. 6).

### ABI

**Fig. 7:**
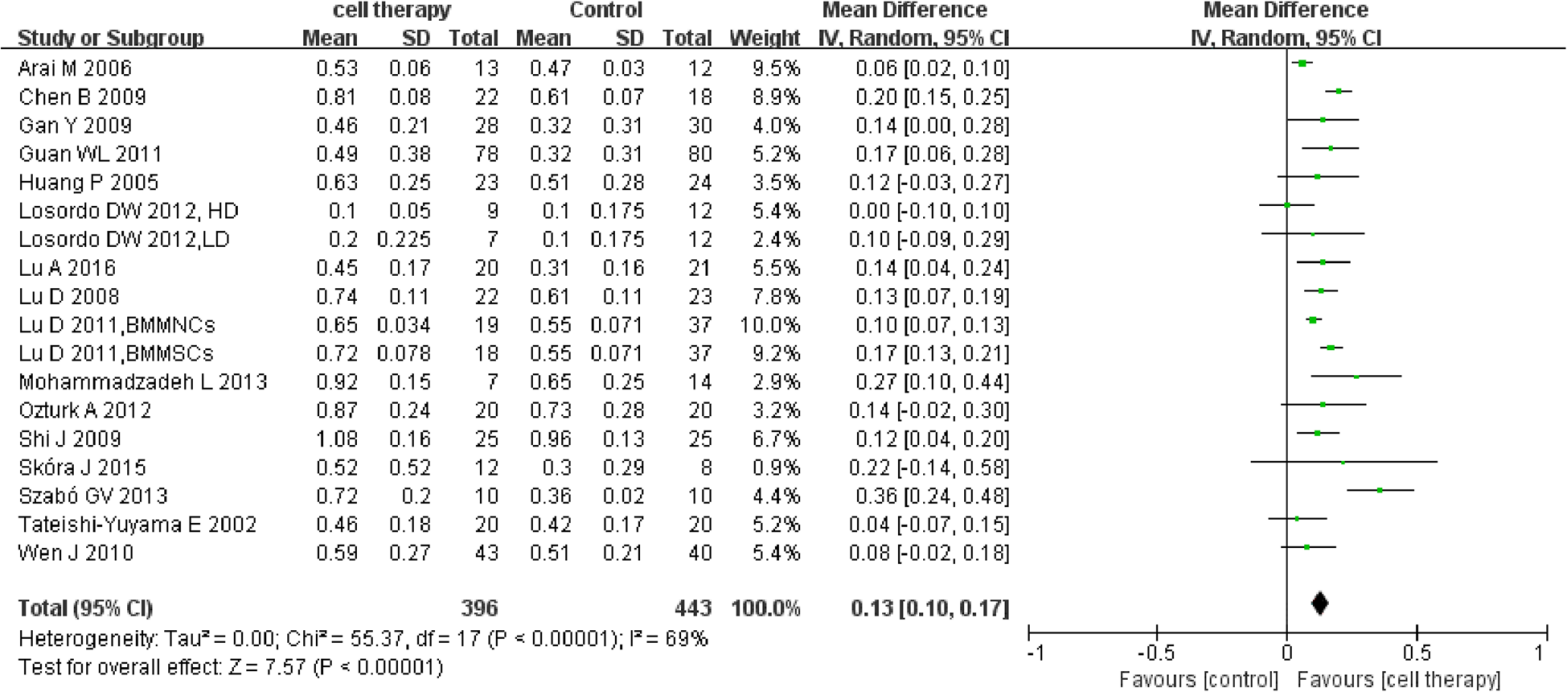
Forest plot showing the effect of stem cell therapy on ABI

Sixteen studies [22, 27, 29, 31, 33–35, 37, 43, 44, 46, 47, 49, 50, 53, 54] reported the detailed ABI. The meta-analysis showed higher ABI in the cell therapy group compared with control (MD 0.13, 95% CI 0.10 to 0.17, *I*^2^ = 69%) (Fig. 7).

### TcO_2_

**Fig. 8:**
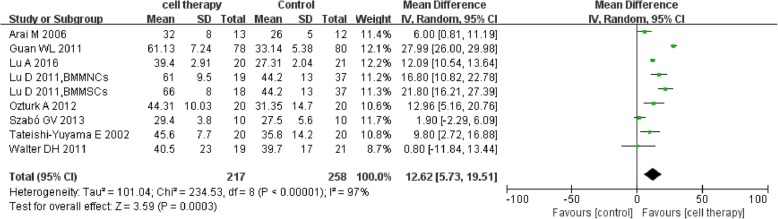
Forest plot showing the effect of stem cell therapy on TcO_2_

Eight studies [22, 29, 38, 43, 44, 46, 49, 54] reported the detailed TcO_2_. The meta-analysis showed higher TcO_2_ in the cell therapy group compared with control (MD 12.62, 95% CI 5.73to 19.51, *I*^2^ = 97%) (Fig. 8).

### Rest pain score

**Fig. 9:**
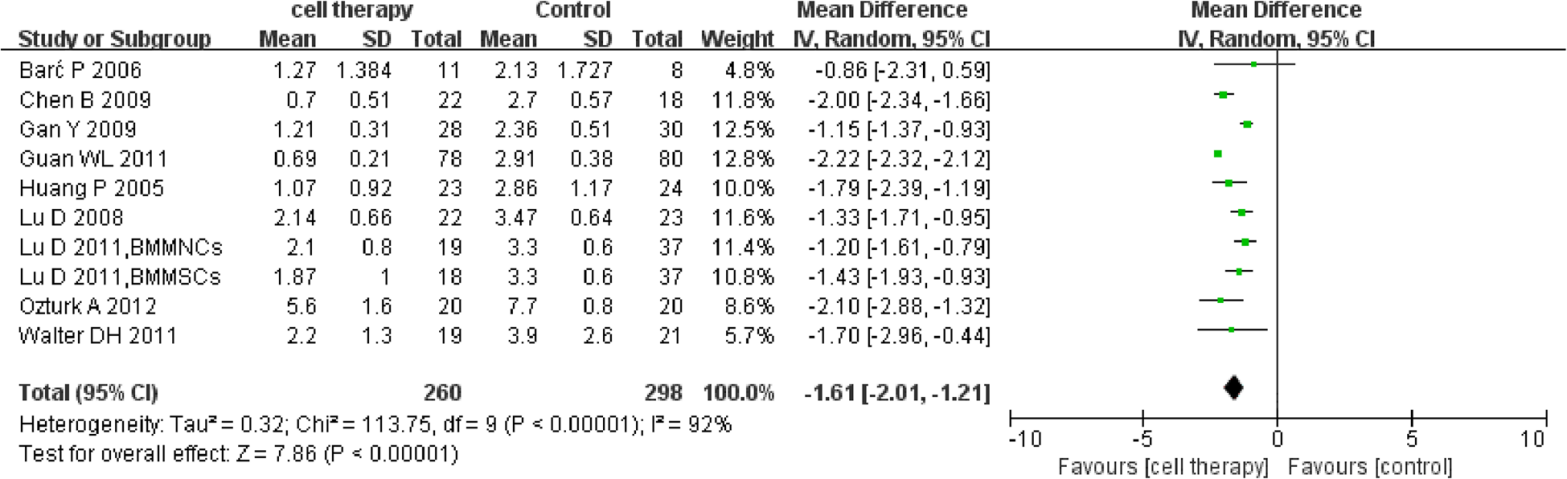
Forest plot showing the effect of stem cell therapy on rest pain score

Nine studies [27, 28, 31, 33, 34, 38, 43, 44, 46] reported the detailed rest pain score. The meta-analysis showed lower rest pain score in the cell therapy group compared with control (MD − 1.61, 95% CI − 2.01 to − 1.21, *I*^2^ = 92%) (Fig. 9).

### Pain-free walking distance

**Fig. 10:**
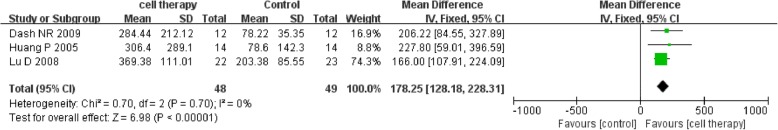
Forest plot showing the effect of stem cell therapy on PFWD

Only three studies [27, 31, 32] reported detailed PFWD. The meta-analysis showed that PFWD in stem cell therapy group was higher than the control group (MD 178.25, 95% CI 128.18 to 228.31, *I*^2^ = 0%) (Fig. 10).

### Subgroup analysis

Thirteen studies [27, 30–35, 43–46, 50, 54] included DM patients. The meta-analysis showed that stem cell therapy could reduce the amputation rate (3/109 vs 32/155; OR 0.17, 95% CI 0.06 to 0.45, *I*^2^ = 0%) (Additional file 2: Figure S1) and improve the ulcer healing rate (167/305 vs 89/304; OR 4.34, 95% CI 2.96 to 6.38, *I*^2^ = 23%) (Additional file 3: Figure S2) in DM patients.

### Side effect association with cell therapy

Eight studies [22, 33, 37, 40, 42, 43, 47, 52] reported the side effect of stem cell therapy. Side effect included slight edema of limbs, transient increase of serum creatine phosphokinase, bleeding, pain, infection, and cellulitis after puncture or injection, hematocrit, proliferative retinopathy, moderate hypotension, and chest distress during mobilization and severe worsening of CLI in the target leg after injection. The most serious side effect was wound sepsis on the injected leg and with the ending of amputation. The detailed side events were showed in Additional file 4: Table S2.

### Publication bias

The funnel plot and statistical test showed publication bias in amputation rate, major amputation rate, ABI, and no publication bias in ulcer healing rate, TcO_2_, rest pain score, and PFWD (Additional files 5, 6, 7, and 8: Figures S3-S6; Additional file 9: Table S3).

### Sensitivity analyses

Results of sensitivity analyses were showed in Additional file 10: Table S4 and Additional file 11: Table S5. All the effect measures obtained by random effects do not significantly differ from those by the fixed effect model except for major amputation rate. RD derived from the random model differed from that in the fixed model.

### Quality of evidence

GRADE evidence profile is showed in Table 2. All the quality evidence of outcomes were low. The low quality may due to inconsistency, imprecision, and publication bias.

**Table 2:** GRADE evidence profile for the outcomes

Certainty assessment							No. of patients		Effect	Quality of evidence	Importance
No. of studies	Study design	Risk of bias	Inconsistency	Indirectness	Imprecision	Publication bias	Stem cell therapy	Control	(95% CI)		
ABI											
16	RCT	No serious limitations	Serious limitations	No serious limitations	No serious limitations	Yes	396	443	MD 0.13 (0.10, 0.17)	⨁⨁◯◯低	Important
TcO2											
8	RCT	No serious limitations	Serious limitations	No serious limitations	No serious limitations	No	217	258	MD 12.62 (5.73, 19.51)	⨁⨁◯◯低	Important
Major amputation rate											
8	RCT	No serious limitations	No serious limitations	No serious limitations	Serious limitations	Yes	49/232 (21.1%)	60/197 (305%)	OR 0.66 (0.42, 1.03)	⨁⨁◯◯低	Key
Amputation rate											
16	RCT	No serious limitations	No serious limitations	No serious limitations	Serious limitations	Yes	88/425 (20.7%)	142/444 (32.0%)	OR 0.50 [0.36, 0.69]	⨁⨁◯◯低	Key
Ulcer healing rate											
14	RCT	No serious limitations	No serious limitations	No serious limitations	Serious limitations	No	170/313 (54.3%)	90/310 (29.0%)	OR 4.31 [2.94, 6.30]	⨁⨁◯◯低	Key
Rest pain score											
9	RCT	No serious limitations	Serious limitations	No serious limitations	No serious limitations	No	260	298	MD − 1.61 [− 2.01, − 1.21]	⨁⨁◯◯低	Important
Pain-free walking distance											
3	RCT	No serious limitations	No serious limitations	No serious limitations	Serious limitations	No	48	49	MD 178.25 [128.18, 228.31]	⨁⨁◯◯低	Important

## Discussion

This meta-analysis indicated that autologous implantation of stem cells improved ulcer healing rate, ABI, TcO2, PFWD, and reduced amputation rate and rest pain score compared with standard care/conventional treatment. Stem cell therapy could reduce major amputation rate but with no statistical significance and seemingly no significant improvement in limb salvage (*P* = 0.64). Sensitivity analysis showed instability in the result of major amputation rate which may be related to small sample size and publication bias. Stem cell therapy could reduce amputation rate and improve ulcer healing rate in DM subgroup. The results suggested that stem cell therapy may alter the outcome of intractable CLI to a certain degree.

To our knowledge, this is the systematic review including the most RCTs of autologous implantation of stem cells for PAD up to now. We excluded one study [55] included in the previous systematic review [23]. The study used allogeneic bone marrow-derived mesenchymal stem cell for implantation, which did not meet the inclusion criteria. But we included nine studies that were not analyzed in the previous systematic review. The study of Tateishi-Yuyama reported two parts of the experiment and one is RCT [22]. The other eight studies [30, 33–35, 37, 44, 45, 53] also met the inclusion criteria in every way but were not included in the previous systematic review. In addition, the previous systematic reviews did secondary analysis including non-RCTs and RCTs, but studies of different designs should not be analyzed in a combined manner. In this case, we believe that our results are more reliable than the previous ones. Besides, we are the first to perform the subgroup analysis for patients with DM who bear the increased risk of PAD, segmental and diffuse arterial disease, and cardiovascular event. Most DM patients are not suitable for surgery or interventional therapy, and they may benefit from stem cell therapy.

Our study showed only one serious side effect related to the implantation of stem cells which shall remind us of the importance of aseptic technique during the injection. Due to the short follow-up, a full understanding of the side effect of stem cell implantation calls for further study. There were some observational studies reporting a serious side effect of stem cell therapy. Horie has reported heart failure, myocardial infarction, severe infection, and stroke post-cell therapy [56]. Moreover, the relationship between the tumor and stem cell therapy remains disputable. Among the 162 patients receiving stem cell implantation in Horie’s study [56], 9 patients had malignant tumor during 24.6 months follow-up. Two patients were diagnosed with a malignant tumor before the study, and the other 7 patients developed a small intestinal tumor, pancreatic cancer, lung cancer, gallbladder carcinoma, gastric cancer, and groin tumor. But this was an observational study and there is no direct cause-and-effect relationship between those events and stem cells therapy. Thus, RCTs of large sample size and longer follow-up time are needed to verify the safety of cell therapy.

There are several limitations in our study. Firstly, most trials have a high or unclear risk of bias so the trials may be underpowered. Low quality of methodology mainly results from inadequate sequence generation, lack of allocation concealment, absent blinding, and incomplete outcome data. Some RCTs mentioned “random” but did not report the specific randomization method. Some RCTs did not use allocation concealment and blinding method. Secondly, several studies had a small sample size and limited information for outcomes, such as adverse events. Thirdly, the included patients, types of stem cells, methods of transplantation, control group, and follow-up time were different among RCTs, which may lead to heterogeneity. The patients in the included studies were identified as having PAD or DF according to a different classification. There were eight types of stem cells including BMMSCs, BMMNCs, BMAC, PBMNC, CD34+ cells, VesCell, PBMCs, and CD133+ cells in our studies. Stem cells were transplanted by intramuscular injection or intra-arterial injection. Besides, the number of stem cells used varied among RCTs and part of studies did not report the number of transplanted stem cells. Stem cells used in the included studies may be the major cause of heterogeneity. Thus, standardization in the transplantation method, stem cell type, and quantity should be valued in transplantation. Treatments in control groups contain non-mobilized peripheral blood mononuclear cells, conventional treatment, and placebo. Follow-up time ranged from 1 to 36 months. These differences lead to great heterogeneity in meta-analysis of ABI, TcO2 and rest pain score. Twenty-seven RCTs included in this study all reported positive results, and we only included studies in English and Chinese, which may lead to publication bias.

## Conclusions

The “no-option” patients with PAD may benefit from stem cells therapy, but there was seemingly no significant improvement in major limb salvage. Due to the low-quality evidence, further researches including larger, randomized, double-blinded, placebo-controlled, multicenter trials with long-term follow-up of high quality are still in demand to prove the efficacy and safety of stem cells therapy for PAD.

## Additional files

Additional file 1: **Table S1.** Details of search terms. (DOCX 13 kb)

Additional file 2: **Figure S1.** Forest plot showing the effect of stem cell therapy on amputation rate in DM subgroup. (DOCX 24 kb)

Additional file 3: **Figure S2.** Forest plot showing the effect of stem cell therapy on ulcer healing rate in DM subgroup. (DOCX 27 kb)

Additional file 4: **Table S2.** Side effect association with stem cell therapy. (DOCX 16 kb)

Additional file 5: **Figure S3.** Funnel plot of amputation rate. (DOCX 22 kb)

Additional file 6: **Figure S4.** Funnel plot of ulcer healing rate. (DOCX 22 kb)

Additional file 7: **Figure S5.** Funnel plot of ABI. (DOCX 21 kb)

Additional file 8: **Figure S6.** Funnel plot of rest pain score. (DOCX 21 kb)

Additional file 9: **Table S3.** Statistical test showed publication bias. (DOCX 15 kb)

Additional file 10: **Table S4.** Sensitivity analysis: random model VS fixed model and OR VS RR VS RD on outcomes (*P > 0.05). (DOCX 15 kb)

Additional file 11: **Table S5.** Sensitivity analysis: random model VS fixed model and MD VS SMD on outcome. (DOCX 15 kb)

## Abbreviations

ABI: Ankle-brachial index
CENTRAL: Cochrane Central Register of Controlled Trials
CI: Confidence intervals
CLI: Critical limb ischemia
CNKI: China National Knowledge Infrastructure
DF: Diabetic foot
DM: Diabetes mellitus
EPC: Endothelial progenitor cell
MD: Mean difference
OR: Odds ratios
PAD: Peripheral arterial disease
PFWD: Pain-free walking distance
PRISMA: Preferred reporting items for systematic reviews and meta-analyses
RCT: Randomized controlled trial
RD: Risk difference
RR: Relative risk
SMD: Standard mean difference
TACT: Therapeutic angiogenesis using cell transplantation
TcO_2_: Transcutaneous oxygen tension
