# Supplementary material for: Autologous stem cell therapy for peripheral arterial disease: a systematic review and meta-analysis of randomized controlled trials
Source: Stem Cell Res Ther. 2019 May 21;10:140. doi: 10.1186/s13287-019-1254-5 (PMC6528204; doi:10.1186/s13287-019-1254-5)
Supplement: Supplementary file 1 — Table S1. Details of search terms. (DOCX 13 kb) [file 13287_2019_1254_MOESM1_ESM.docx]

**Additional file 1:Table S1.Details of Search Terms**

| **Search terms used** |
| --- |
| stem cell" OR "mononuclear cell" OR "bone marrow" OR "granulocyte colony stimulating factor" OR" cell therapy" OR " G-CSF "OR"CD34+"OR"CD133+"AND " peripheral arterial disease " OR " PAD " OR" critical limb ischemia " OR " CLI " OR " peripheral vascular disease " OR "diabetic foot "OR" obliterans" |
