# Supplementary material for: Autologous stem cell therapy for peripheral arterial disease: a systematic review and meta-analysis of randomized controlled trials
Source: Stem Cell Res Ther. 2019 May 21;10:140. doi: 10.1186/s13287-019-1254-5 (PMC6528204; doi:10.1186/s13287-019-1254-5)

**Additional file 3:Figure S2．Forest plot showing eﬀect of stem cell therapy on ulcer healing rate in DM subgroup**


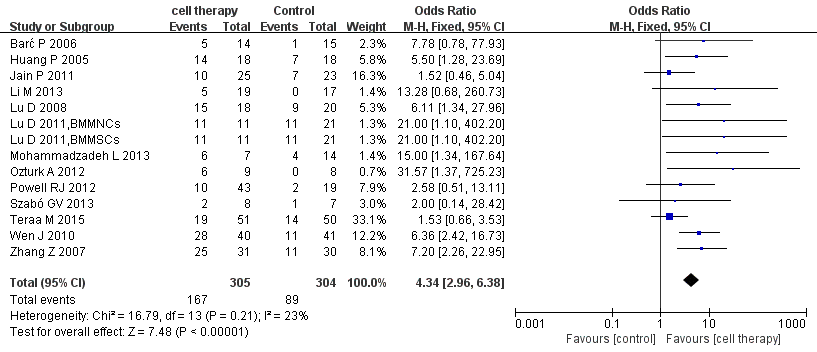

Supplement: Supplementary file 3 — Figure S2. Forest plot showing the effect of stem cell therapy on ulcer healing rate in DM subgroup. (DOCX 27 kb) [file 13287_2019_1254_MOESM3_ESM.docx]
