# Supplementary material for: Autologous stem cell therapy for peripheral arterial disease: a systematic review and meta-analysis of randomized controlled trials
Source: Stem Cell Res Ther. 2019 May 21;10:140. doi: 10.1186/s13287-019-1254-5 (PMC6528204; doi:10.1186/s13287-019-1254-5)
Supplement: Supplementary file 4 — Table S2. Side effect association with stem cell therapy. (DOCX 16 kb) [file 13287_2019_1254_MOESM4_ESM.docx]

**Additional file 4:Table S2. Side effect association with stem cell therapy**

| Study(year) | Side effect | Prognosis |
| --- | --- | --- |
| Tateishi-Yuyama E,（2002）^[22]^ | Transient and slight increase of concentrations of serum creatine phosphokinase | Returned to normal |
| Chen B（2009）^[33]^ | Slight limb swell | Improved |
| Wen J(2010)^[37]^ | Two patients with coronary heart disease had chest distress during mobilization | Improved |
| BenoitE(2011)^[40]^ | Rransient and mean decrease in hematocrit, proliferative retinopathy in 4 patients | No worsened retinopathy |
| PowellRJ(2012)^[42]^ | Injection site pain or pain in extremity or foot in 5 patients, one patient developed wound sepsis on the first toe of the injected leg, cellulitis in one patient, eczema in one patient, thrombophlebitis in one patient | Amputation(patientwith sepsis),unclear(other patient) |
| Lu D(2011)^[43]^ | Three patients bled at the iliac crest, 5 limbs had slight pain | Improved |
| [Losordo DW](https://www.ncbi.nlm.nih.gov/pubmed/?term=Losordo%20DW%5BAuthor%5D&cauthor=true&cauthor_uid=23192920)，(2012)^[47]^ | One patient experienced moderate hypotension, one patient experienced severe worsening of CLI | Improved |
| Teraa M(2015)^[52]^ | An inguinal hematoma in one patient | Unclear |
