# Supplementary figures and images for: Autologous stem cell therapy for peripheral arterial disease: a systematic review and meta-analysis of randomized controlled trials
Source: Stem Cell Res Ther. 2019 May 21;10:140. doi: 10.1186/s13287-019-1254-5 (PMC6528204; doi:10.1186/s13287-019-1254-5)

**Additional file 5:Figure S3．Funnel plot of amputation rate**


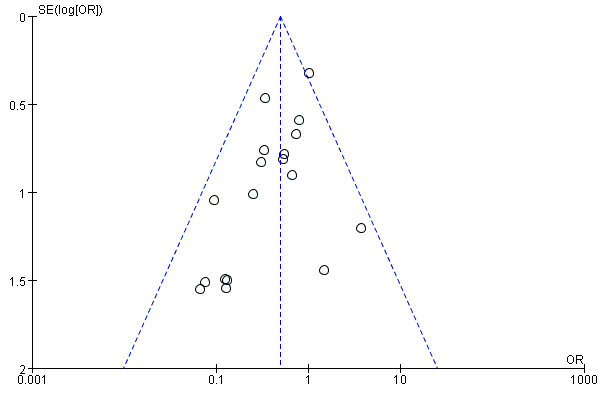

Supplement: Supplementary file 5 — Figure S3. Funnel plot of amputation rate. (DOCX 22 kb) [file 13287_2019_1254_MOESM5_ESM.docx]

**Additional file 6:Figure S4．Funnel plot of ulcer healing rate**


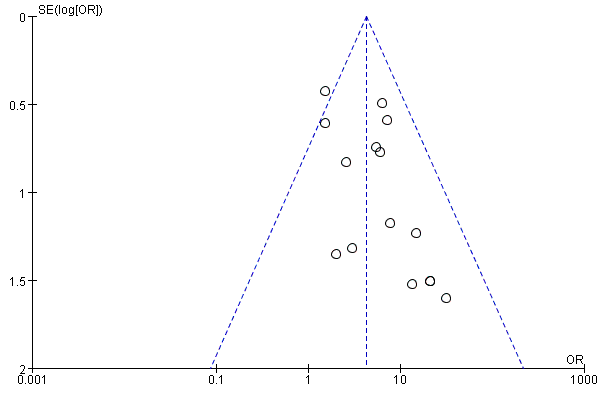

Supplement: Supplementary file 6 — Figure S4. Funnel plot of ulcer healing rate. (DOCX 22 kb) [file 13287_2019_1254_MOESM6_ESM.docx]

**Additional file 7:Figure S5．Funnel plot of ABI**


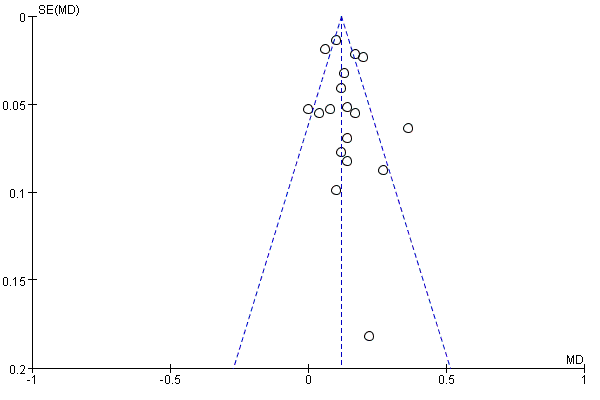

Supplement: Supplementary file 7 — Figure S5. Funnel plot of ABI. (DOCX 21 kb) [file 13287_2019_1254_MOESM7_ESM.docx]

**Additional file 8:Figure S6．Funnel plot of rest pain score**


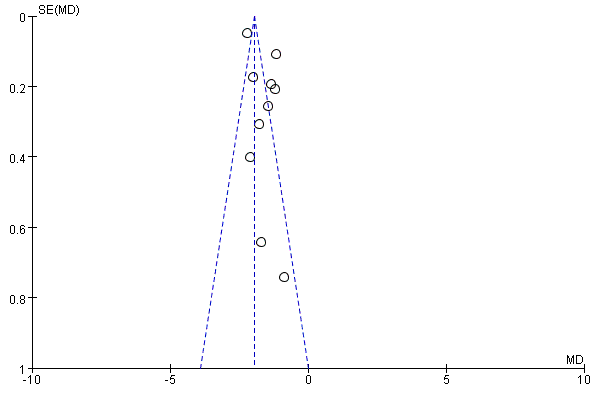

Supplement: Supplementary file 8 — Figure S6. Funnel plot of rest pain score. (DOCX 21 kb) [file 13287_2019_1254_MOESM8_ESM.docx]
