# Supplementary material for: Autologous stem cell therapy for peripheral arterial disease: a systematic review and meta-analysis of randomized controlled trials
Source: Stem Cell Res Ther. 2019 May 21;10:140. doi: 10.1186/s13287-019-1254-5 (PMC6528204; doi:10.1186/s13287-019-1254-5)
Supplement: Supplementary file 9 — Table S3. Statistical test showed publication bias. (DOCX 15 kb) [file 13287_2019_1254_MOESM9_ESM.docx]

**Additional file 9:Table S3.** Statistical test showed publication bias

| Effect measure | Amputation rate | Major amputation rate | Ulcer healing rate | ABI | TcO_2_ | Rest pain score | Pain-free walking distance |
| --- | --- | --- | --- | --- | --- | --- | --- |
| Statistical approach | Harbord’s test | Harbord’s test | Harbord’s test | Egger’s test | Egger’s test | Egger’s test | Egger’s test |
| P | 0.057 | 0.012 | 0.178 | 0.037 | 0.887 | 0.193 | 0.613 |
