# Supplementary material for: Autologous stem cell therapy for peripheral arterial disease: a systematic review and meta-analysis of randomized controlled trials
Source: Stem Cell Res Ther. 2019 May 21;10:140. doi: 10.1186/s13287-019-1254-5 (PMC6528204; doi:10.1186/s13287-019-1254-5)
Supplement: Supplementary file 10 — Table S4. Sensitivity analysis: random model VS fixed model and OR VS RR VS RD on outcomes (*P > 0.05). (DOCX 15 kb) [file 13287_2019_1254_MOESM10_ESM.docx]

**Additional file 10:Table S4.** **Sensitivity Analysis: random model VS fixed model and OR VS RR VS RD on outcomes**

|  |  | Amputation rate | Major amputation rate | Ulcer healing rate |
| --- | --- | --- | --- | --- |
| OR | Random model | 0.50 [0.34, 0.74] | 0.69 [0.43, 1.10]* | 4.32 [2.72, 6.87] |
|  | Fixed model | 0.50 [0.36, 0.69] | 0.66 [0.42, 1.03]* | 4.31 [2.94, 6.30] |
| RR | Random model | 0.67 [0.52, 0.87] | 0.77 [0.55, 1.08]* | 1.96 [1.64, 2.33] |
|  | Fixed model | 0.62 [0.49, 0.78] | 0.74 [0.53, 1.03]* | 2.07 [1.71, 2.51] |
| RD | Random model | -0.14 [-0.20, -0.07] | -0.12 [-0.23, -0.01] | 0.33 [0.24, 0.42] |
|  | Fixed model | -0.12 [-0.18, -0.07] | -0.08 [-0.16, -0.00]* | 0.30 [0.24, 0.37] |

*P>0.05
