# Supplementary material for: Autologous stem cell therapy for peripheral arterial disease: a systematic review and meta-analysis of randomized controlled trials
Source: Stem Cell Res Ther. 2019 May 21;10:140. doi: 10.1186/s13287-019-1254-5 (PMC6528204; doi:10.1186/s13287-019-1254-5)
Supplement: Supplementary file 11 — Table S5. Sensitivity analysis: random model VS fixed model and MD VS SMD on outcome. (DOCX 15 kb) [file 13287_2019_1254_MOESM11_ESM.docx]

**Additional file 11:Table S5. Sensitivity Analysis: Sensitivity Analysis: random model VS fixed model and MD VS SMD on outcomes**

|  |  | ABI | TcO2 | Rest pain score | Pain-free walking distance |
| --- | --- | --- | --- | --- | --- |
| MD | Random model | 0.13 [0.10, 0.17] | 12.62[5.73, 19.51] | -1.61 [-2.01, -1.21] | 178.25 [128.18, 228.31] |
|  | Fixed model | 0.12 [0.11, 0.14] | 16.17 [15.10, 17.24] | -1.96 [-2.04, -1.88] | 178.25 [128.18, 228.31] |
| SMD | Random model | 0.92 [0.61, 1.23] | 1.69 [0.65, 2.74] | -2.36 [-3.40, -1.33] | 1.35 [0.90, 1.79] |
|  | Fxed model | \| 0.77 [0.63,0.92] \| \| --- \| | 1.62 [1.39, 1.86] | -2.16 [-2.39, -1.93] | 1.35 [0.90, 1.79] |
